# The methionine synthase polymorphism D919G alters susceptibility to primary central nervous system lymphoma

**DOI:** 10.1038/sj.bjc.6601777

**Published:** 2004-04-06

**Authors:** M Linnebank, S Schmidt, H Kolsch, A Linnebank, R Heun, I G H Schmidt-Wolf, A Glasmacher, K Fliessbach, T Klockgether, U Schlegel, H Pels

**Affiliations:** 1 Department of Neurology, University Hospital of Bonn, Sigmund-Freud-Str. 25, Bonn D-53125, Germany; 2 Department of Psychiatry, University Hospital of Bonn, Sigmund-Freud-Str. 25, Bonn D-53125, Germany; 3 Department of Internal Medicine, University Hospital of Bonn, Sigmund-Freud-Str. 25, Bonn D-53105, Germany

**Keywords:** PCNSL, methionine synthase, cystathionine beta-synthase, MTHFR, homocysteine, folate

## Abstract

Primary central nervous system lymphomas (PCNSL) frequently reveal genomic instability. We analysed different functional genetic variants affecting the folate and homocysteine metabolism important for DNA integrity in 31 PCNSL patients and 142 controls. We found significantly less carriers of the methionine synthase c.2756A>G (D919G) missense polymorphism among the patients (0.16 *vs* 0.42; odds ratio 0.26, CI_95%_: 0.09–0.74; *P*=0.005), suggesting a protective function of the G allele. These data stimulate further epidemiological and functional studies focusing on the role of homocysteine and folate metabolism in lymphoma tumorigenesis.

Primary central nervous system lymphomas (PCNSL) are highly malignant non-Hodgkin's lymphomas (NHL). The vast majority of them are of the diffuse large B-cell type (Montesinos-Rongen *et al*, 1999; DLBCL; Garter and Warnke, 2001). Genetic instability with characteristic chromosomal imbalances is a characteristic feature of PCNSL (Weber *et al*, 2000; Gonzalez-Gomez *et al*, 2003; Nakamura *et al*, 2003). Since homocysteine and folate metabolism is closely linked to DNA methylation, it considerably contributes to the maintenance of DNA integrity (Figure 1

**Figure 1:**
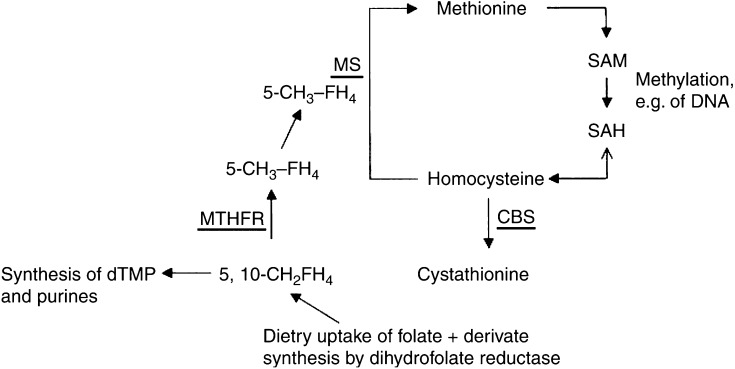
Folate and homocysteine metabolism in human. In human homocysteine metabolism, methionine is activated to SAM. *S*-adenosylmethionine is the ubiquitous methyl group donor, for example, for DNA methylation. Demethylation of SAM results in *S*-adenosylhomocysteine (SAH), which is hydrolysed to homocysteine. Homocysteine can be trans-sulphurated to cystathionine by CBS or remethylated to methionine by MS. Methionine synthase needs 5-methyltetrahydrofolate (5-CH_3_-FH_4_) as cofactor, which is generated from 5,10-methylenetetrahydrofolate (5,10-CH_2_FH_4_) by MTHFR. Competitively, 5,10-CH_2_FH_4_ is used for nucleic acid synthesis (dTMP and purines).

; Mudd *et al*, 2001). Consequently, genetic variants that functionally influence homocysteine and folate metabolism are associated with different types of cancer, for example, systemic NHL, acute leukaemia and colorectal cancer (Ma *et al*, 1997; Skibola *et al*, 1999; Matsuo *et al*, 2001; Gonzalez-Fraile *et al*, 2002; Lincz *et al*, 2003). To test the hypothesis that genetic variants of the homocysteine and folate metabolism alter the susceptibility of developing PCNSL, we analysed five polymorphisms affecting both the availability of 5,10-methylenetetrahydrofolate as a cosubstrate in nucleic acid synthesis and the level of *S*-adenosylmethionine (SAM) as a cosubstrate in DNA methylation.

## MATERIALS AND METHODS

We performed a case–control study using DNA samples of 31 German patients with PCNSL of the DLBCL-subtype (m/f: 11/20; age: 61±10 years; Pels *et al*, 2003). DNA samples of 142 healthy German individuals (m/f: 62/80; age: 63±14 years), matched for the area of residence, served as controls. Genomic DNA was prepared according to standard methods (Miller *et al*, 1988). Five functional polymorphisms were analysed as previously described: Cystathionin beta-synthase (CBS) c.833T>C (I278 T) and 844ins68 (splice variant), methionine synthase (MS) c.2756A>G (D919G), and methylenetetrahydrofolate reductase (MTHFR) c.677C>T (A222 V) and c.1298A>C (E429A). With the exception of CBS I278 T, these polymorphisms have a high prevalence within the healthy German population with allele frequencies ranging between 0.08 and 0.34 (Harmon *et al*, 1999; Linnebank *et al*, 2000, 2001).

Logistic regression analysis was used to examine the association of single or combined polymorphisms with PCNSL and to exclude confounding effects of age, gender or multiple testing. In addition, *χ*^2^-tests were used to test differences of allele frequencies in patients and controls. Threshold was defined with *P*<0.05. The study was approved by the local ethics committee, and all participants gave written informed consent.

## RESULTS

The Hardy–Weinberg equilibrium was confirmed for all polymorphisms. Allele frequencies of the control group did not significantly differ from those reported in the literature (Harmon *et al*, 1999; Linnebank *et al*, 2000, 2001; Table 1

**Table 1:** Genotypes in PCNSL patients and controls: *χ*^2^-analysis

**CBSc.833**	**TT**	**CT**	**CC**	**T-alleles**	**C-alleles**	***χ*^2^ (df=1)**	***P* (*χ*^2^)**
PCNSL (*n*=31)	31 (1.00)	0	0	62 (1.00)	0	0.89	0.35
Controls (*n*=142)	138 (0.97)	4 (0.03)	0	280 (0.99)	4 (0.01)		
							
**CBS 844ins68**	**del/del**	**del/ins**	**ins/ins**	**del-alleles**	**ins-alleles**	***χ*^2^**	***P***
PCNSL (*n*=31)	23 (0.74)	8 (0.26)	0	54 (0.87)	8 (0.13)	2.56	0.09^*^
Controls (*n*=142)	122 (0.86)	20 (0.14)	0	264 (0.93)	20 (0.07)		
							
**MS c.2756**	**AA**	**AG**	**GG**	**A-alleles**	**G-alleles**	***χ*^2^**	***P***
PCNSL (*n*=31)	26 (0.84)	5 (0.16)	0	57 (0.92)	5 (0.08)	7.27	0.007*
Controls (*n*=142)	83 (0.59)	51 (0.36)	8 (0.06)	217 (0.76)	67 (0.24)		
							
**MTHFR c.677**	**CC**	**CT**	**TT**	**C-alleles**	**T-alleles**	***χ*^2^**	***P***
PCNSL (*n*=31)	13 (0.42)	12 (0.39)	6 (0.19)	38 (0.61)	24 (0.39)	0.23	0.64
Controls (*n*=142)	66 (0.47)	52 (0.37)	24 (0.17)	184 (0.65)	100 (0.35)		
							
**MTHFR c.1298**	**AA**	**AC**	**CC**	**A-alleles**	**C-alleles**	***χ*^2^**	***P***
PCNSL (*n*=31)	16 (0.52)	12 (0.39)	3 (0.10)	44 (0.71)	18 (0.29)	0.24	0.63
Controls (*n*=142)	69 (0.49)	54 (0.38)	19 (0.13)	192 (0.68)	92 (0.32)		

* Results from logistic regression (as given in the text): *P*=0.005 for MS c.2756A>C and *P*=0.08 for CBS 844ins68.

). The MS c.2756A>G polymorphism (G-allele) was significantly less frequent in patients than in controls. In all, 6% of controls, but none of the patients were homozygous for the G-allele. At least one mutant allele was detected in 16% of PCNSL patients compared to 42% of controls (odds ratio (OR): 0.26, CI_95%_: 0.09 – 0.74; *P*=0.005 in regression analysis; *P*=0.007 in uncorrected *χ*^2^-analysis; *P*=0.035 in *χ*^2^-analysis corrected for multiple testing).

The CBS 844ins68 polymorphism was detected more frequently in patients (26%) than in controls (14%). However, the difference did not reach statistical significance in regression analysis (OR: 2.43, CI_95%_: 0.90 – 6.61; *P*=0.08) or *χ*^2^-analysis (*P*=0.09, uncorrected). Further, we did not find any significant association of the other polymorphisms (MTHFR c.677C>T or c.1298A>C, CBS c.833T>C) or combinations with PCNSL.

## DISCUSSION

In the present study, we investigated the association of functional variants of the homocysteine and folate metabolism with PCNSL. Previous studies found that the MS missense dimorphism c.2756A>G (D919G) is associated with a lower risk of developing colorectal cancer and types of systemic NHL (Ma *et al*, 1999; Lincz *et al*, 2003). Our findings suggest a protective function of MS c.2756A>G against PCNSL.

Methionine synthase c.2756A>G reduces MS activity (Harmon *et al*, 1999) leading to a lower consumption of 5,10- methylenetetrahydrofolate by the homocysteine metabolism. As a consequence, increased levels of 5,10-methylenetetrahydrofolate are available for nucleic acid synthesis, thus reducing the risk of thymidin deprivation and uracil misincorporation associated with chromosomal breakage (Blount *et al*, 1997; Harmon *et al*, 1999; Figure 1). Moreover, reduced MS activity due to MS c.2756A>G results in lower rates of synthesis of methionine and SAM, which might be protective against SAM-dependent DNA hypermethylation that is an additional risk factor for cancer development (Figure 1; Matsuo *et al*, 2001; Mudd *et al*, 2001): several studies have demonstrated an association of DNA hypermethylation of tumour-suppressor genes such as p53, p15 and p16 with lymphomas (Baur *et al*, 1999; Moller *et al*, 1999; Gonzalez *et al*, 2000).

The functional consequences of CBS 844ins68 are thought to be opposite to that of MS c.2756A>G. 844ins68 lowers CBS activity leading to an increase of homocysteine remethylation and reduced availability of 5,10-methylentetrahydrofolate for nucleic acid synthesis, while synthesis of SAM that serves as the substrate for DNA methylation is increased (Mudd *et at*, 2001; Figure 1). We found a higher frequency of CBS844ins68 in PCNSL patients than in controls. However, this difference did not reach statistical significance.

The MTHFR variants c.677C>T and c.1298A>C and their combinations have previously been reported to be associated with several types of human cancer (Ma *et al*, 1997; Skibola *et al*, 1999; Matsuo *et al*, 2001), but there were no significant associations of these polymorphisms with PCNSL in our study.

In summary, the results of this study suggest a protective function of MS c.2756A>G (G allele) against PCNSL and encourage future studies investigating the influence of genetic and dietary conditions of homocysteine and folate metabolism on cancerogenesis.
